# Inside-out chicken enteroids with leukocyte component as a model to study host–pathogen interactions

**DOI:** 10.1038/s42003-021-01901-z

**Published:** 2021-03-19

**Authors:** Tessa J. Nash, Katrina M. Morris, Neil A. Mabbott, Lonneke Vervelde

**Affiliations:** Division of Infection and Immunity, The Roslin Institute and Royal (Dick) School of Veterinary Studies, University of Edinburgh, Midlothian, UK

**Keywords:** Antimicrobial responses, Intestinal stem cells

## Abstract

Mammalian three-dimensional (3D) enteroids mirror in vivo intestinal organisation and are powerful tools to investigate intestinal cell biology and host–pathogen interactions. We have developed complex multilobulated 3D chicken enteroids from intestinal embryonic villi and adult crypts. These avian enteroids develop optimally in suspension without the structural support required to produce mammalian enteroids, resulting in an inside-out enteroid conformation with media-facing apical brush borders. Histological and transcriptional analyses show these enteroids comprise of differentiated intestinal epithelial cells bound by cell-cell junctions, and notably, include intraepithelial leukocytes and an inner core of lamina propria leukocytes. The advantageous polarisation of these enteroids has enabled infection of the epithelial apical surface with *Salmonella* Typhimurium, influenza A virus and *Eimeria tenella* without the need for micro-injection. We have created a comprehensive model of the chicken intestine which has the potential to explore epithelial and leukocyte interactions and responses in host–pathogen, food science and pharmaceutical research.

## Abstract (web summary)

Nash et al. develop and characterise multilobulated 3D chicken enteroids from intestinal embryonic villi and adult crypts, which constitute an accessible model of the chicken intestine. This model enables manipulations, such as viral infection, to be performed without the need for micro-injection, thus opening up more opportunities to explore epithelial and leukocyte interactions and responses in host–pathogen, food science and pharmaceutical research.

## Introduction

The expertise to grow continuously expanding self-organising enteroids from adult stem cells has been developed in various mammalian species^1–4^. By isolating intestinal crypts or single multipotent adult stem cells, embedding them in a gel scaffold (often Matrigel, an Engelbreth-Holm-Swarm tumour extract), and adding externally defined growth factors such as epidermal growth factor (EGF), R-spondin and Noggin, 3D enteroids can be generated with organized crypt and villus domains containing many of the differentiated cell types encountered in the gut epithelium in vivo^1^. Typically, the enteroid’s crypts feed into a central functional lumen and are lined by highly polarized epithelial cells whose apical brush borders face the lumen. The basolateral surfaces, in contrast, lie in contact with the extra-cellular matrix (ECM) scaffold^1^. A practical limitation of the 3D geometry and internal lumen of classical enteroids is that they prevent easy access to the apical epithelium. To ensure interaction with the natural site of infection, pathogens are most commonly microinjected into the enteroid lumen^5^, added to disassociated enteroids^6^ or applied to the apical surfaces of 2D enteroid cultures^7^. In addition, mammalian enteroids are generally epithelial in nature, lacking components of the immune system. In order to study the complex interactions between intestinal epithelial cells and leukocytes, co-culture systems have been established but these are complicated to develop and do not represent the array of immune populations in vivo^8–10^. Despite these challenges, enteroids offer a physiologically-relevant in vitro model in which to explore tissue responses to microorganisms. The first effective enteroid-pathogen infection was achieved by Finkbeiner et al. (2012)^6^ who propagated rotavirus within human intestinal organoids. Since then various bacteria^11^, parasites^12,13^ and viruses^6,14^ have been successfully utilised in enteroid infections across several mammalian species^15^.

Research in avian species is a global economic imperative. Over the last 20 years global poultry production has tripled with approximately 107 million tonnes of chicken meat and 1.3 trillion eggs now produced every year^16^. Expansion of the industry is predicted to continue for at least 30 years, with most growth in Africa and Asia^17^. Of particular concern are zoonotic infections such as avian influenza virus and salmonellae for their effects on public health, but also protozoal parasites like *Eimeria* which have a significant impact on animal welfare and the economy^18^. In vitro avian gastrointestinal studies have long been hampered by the lack of representative intestinal cell culture models. Attempting to grow chicken enteroids in the microenvironments successful for other species have so far yielded limited results, revealing thin-walled structures with few if any defined crypt- and villus-like domains^19–21^. Growth factors that support mammalian enteroid proliferation can positively influence chicken cultures^20–22^, however neither chicken enteroids nor primary intestinal monolayers have been shown to resemble the array of differentiated cells found in the avian in vivo intestine^23,24^.

Here we report the development of avian enteroids with multiple villus-crypt structures that maintain the cellular diversity, polarity and barrier function present within the chicken intestinal epithelium in vivo. Histological and transcriptional analyses show these enteroids contain intestinal stem cells, enterocytes, Paneth cells, goblet cells and enteroendocrine cells. In addition, the natural presence of intra-epithelial and lamina propria leukocytes makes this a distinctive model from their mammalian counterparts. We have identified growth conditions distinct from traditional enteroid cultures, which allow the enteroids to display a reverse architecture where a continuous layer of enterocytes are polarised so the abundant microvilli on their apical surface face the media. To expand the potential applications of this avian culture system we have developed enteroids from several poultry species and from different regions of the small and large intestine. The inside-out phenotype has enabled modelling infection of the enteroids with the important avian pathogens *Salmonella* Typhimurium, influenza A virus, and the Apicomplexan parasite *Eimeria tenella*, without the need for complicated manipulation or microinjection. These unique inside-out, leukocyte-containing, avian enteroids represent a physiologically-relevant in vitro system that can be readily applied to study complex host–pathogen epithelial and leukocyte interactions in the avian gut.

## Results

### Differentiation of multilobulated 3D chicken enteroids

**Fig. 1:**
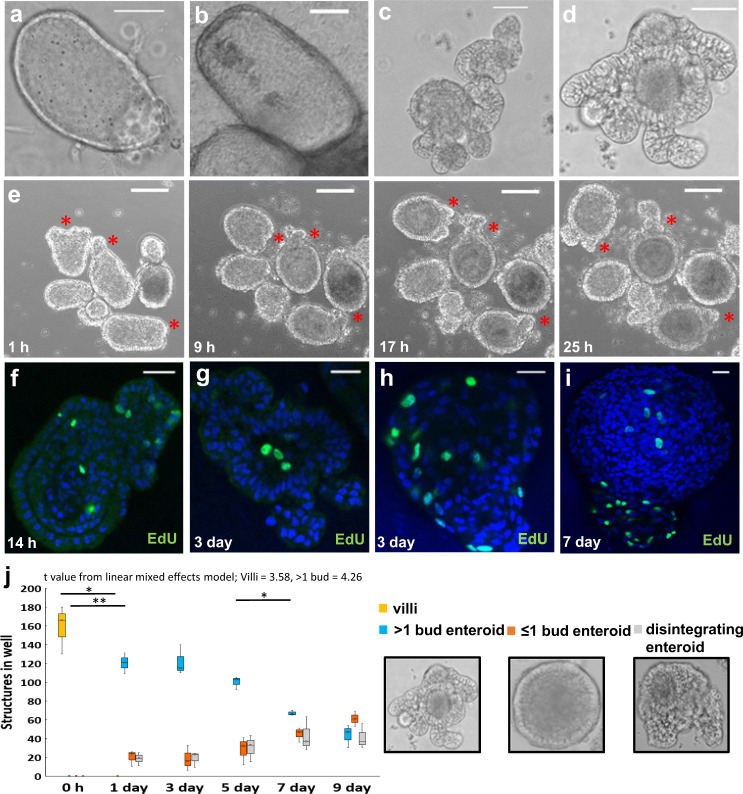
Establishment of floating chicken enteroids. **a** Matrigel-embedded embryonic chicken spheroids increase in size but lack budding at day 1 and **b** day 7 of culture. **c** Floating large multi-lobulated chicken enteroid structures develop over 3 days and **d** are maintained at day 9 of culture. Scale bar: 50 µm. **e** Time-lapse images from Supplementary video 1 showing the formation of budding crypt-like structures (marked by star *) in floating embryonic enteroids. Images are representative of data from at least 20 independent cultures each containing 2–3 embryos. Scale bar: 100 µm. Confocal images of floating embryonic enteroids showing proliferating cells (green, EdU) in both buds and core at **f** 14 h, **g**, **h** 3 days and **i** 7 days of culture. Images are representative of 3 independent cultures. Scale bar: 200 µm. **j** Quantification of floating embryonic enteroid numbers and morphology showed a significant increase in multiple budding enteroid numbers in the first day of culture. Data averaged for 3 cultures containing 2–3 embryos, 3 wells/culture, ~180 villi seeded per well at day 0. Villi: *t = 3.58, *F* = 8.07, df = 1; >1 bud enteroid: *t = 4.26, F = 0.03, df = 1 using a linear mixed effects model. Villi 0 h – 1 day: *p = 0.009, *t* = 10.65; >1 bud enteroid 0 h – 1 day: **p = 0.003, *t* = −18.92; >1 bud enteroid 5–7 day: *p = 0.008, *t* = 10.8 using post hoc pairwise *t*-tests.

Microenvironments successful for mammalian species have so far yielded limited results in birds^19,20^, hence the investigation into alternative stem-cell isolation and enteroid culture techniques. Avian intestinal 18 embryonic day (ED) villi seeded in Matrigel quickly formed spheroid structures that increased in size over 7 days of culture, however the architecture remained basic with no obviously defined crypt-villus development (Fig. 1a, b). By omitting the Matrigel and simply floating the chicken villi/crypts in media alone, enteroid morphology dramatically improved, indicating a component of the matrix is responsible for inhibiting enteroid budding (Fig. 1c, d). Live cell imaging showed isolated tissues rapidly and consistently formed compact spheroid structures by 4 h of culture, and by 24 h multiple budding domains began to establish (Fig. 1e, supplementary Video 1). These growing buds contained numerous proliferating (EdU + ) cells at 14 h of culture (Fig. 1f). By day 2–3, most chicken enteroids had developed numerous elongated buds, which continued to lengthen over the following 1–2 weeks in culture (Fig. 1c, d). Cell proliferation continued to occur in the core and buds at 3 and 7 days of culture (Fig. 1g–i). Multiple budding enteroid numbers increased significantly in the first day of culture then decreased between 5–7 days (Fig. 1j) but the expression of apoptosis markers was relatively stable over the culture period (supplementary Fig. 1).

### Floating poultry enteroids demonstrate an inside-out phenotype

**Fig. 2:**
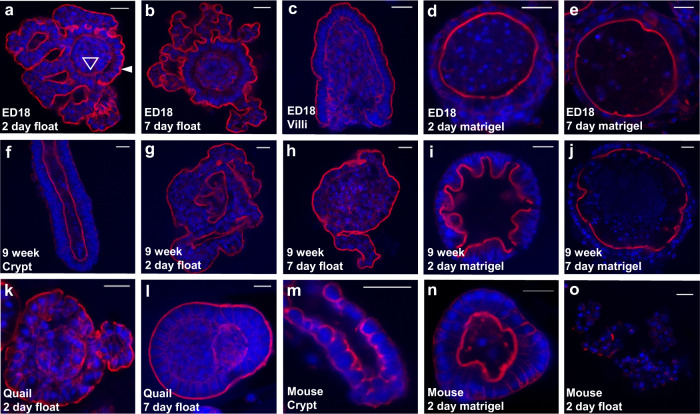
Reverse polarisation of avian floating enteroids. Confocal images of whole-mount enteroids stained to detect F-actin-expressing brush borders (red) and DAPI to visualise cell nuclei (blue). **a** Floating embryonic chicken enteroids at 2 days of culture showing epithelial cells polarised with the apical brush border (closed arrow) facing the media and basal lamina (open arrow) sits on a central core of cells. **b** Embryonic chicken enteroids at 7 days of culture showing ‘inside-out’ polarisation. **c** Tissue isolated from embryonic chicken intestine to form enteroids are villi. **d** Embryonic enteroids cultured in Matrigel for 2 days with epithelial cells polarised so the apical brush border is facing a central lumen. **e** Embryonic enteroids cultured in Matrigel for 7 days. **f** Isolated crypts from 9 week old chicken intestine. **g** Enteroids from 9 week old chickens mimic embryonic chicken enteroid polarisation at 2 days and **h** 7 days in floating culture. **i** Matrigel-embedded enteroids from 9 week old chickens at 2 days and **j** 7 days. **k** Enteroids derived from 1 week old quail also show ‘inside-out’ polarisation at 2 days and **l** 7 days of culture. **m** Isolated crypts from adult mouse intestine. **n** Matrigel-embedded mouse enteroid with internal lumen at 2 days in culture. **o** Floating mouse enteroid at 2 days in culture showing dissociated crypt cells. Scale bar: 20 µm. Images **a**–**e**, and **f**–**o** are representative of 3 cultures composed of 3 chicken embryos and 1 mouse per culture respectively.

Self-organising chicken and mammalian enteroids embedded in Matrigel demonstrate a single sheet of epithelial cells that are polarized so the microvilli surface is facing into a central lumen^1^. In contrast, phalloidin staining to detect F-actin showed that the epithelial cells of embryonic floating chicken enteroids at 2 and 7 days after cultivation had an atypical reversed polarity (Fig. 2a, b). The F-actin positive apical brush border could be visualised on the external epithelial surface facing the media (Fig. 2a closed arrow). The basal surface of the epithelial cells, in contrast, abutted a dense central cellular core (Fig. 2a open arrow). We identified that the isolation procedure from embryonic chicken intestine resulted in collection of villus structures with an external brush border and cell-dense internal structure rather than crypts (Fig. 2c). In contrast, analysis of digested and fractionated adult avian and mouse small intestine showed the expected isolated crypts with an internal dense F-actin positive brush border (Fig. 2f, m).

To confirm the inside-out phenomenon was unique to floating enteroids, the isolated embryonic chicken villi were seeded into Matrigel domes and imaged at 2 and 7 days of culture (Fig. 2d, e). At both time-points orientation of the Matrigel-embedded chicken enteroids reflected their mammalian Matrigel-embedded enteroid counterparts, with the basal cell surface polarised towards the outside, touching the Matrigel, and enterocyte brush borders forming the central luminal surface. The direction of the Matrigel-embedded enteroids’ epithelial apical-basolateral polarity was in stark contrast to that of the floating enteroids (Fig. 2a, b).

Similar staining was performed on 2 and 7 day chicken enteroids derived from 9 week old birds to investigate if the reverse polarity was related to the age and type of progenitor enteroid tissue (Fig. 2f–j). Enteroids developed from the crypts of 6–9 week old chickens displayed the same phenotypes when grown in Matrigel (internal brush border) or floating (external brush border) as enteroids developed from the villi of embryos. In mature birds, the enteroids developed fewer buds and the long-term viability of the cultures was lower compared to those prepared from embryonic tissue (supplementary Fig. 2).

In order to explore whether the development of inside-out enteroids from floating crypts/villi was a species-specific phenomenon, F-actin staining of floating enteroids from 2 day old quail was performed at 2 and 7 days of culture (Fig. 2k, l). The external brush border of quail enteroids mirrored data obtained from chickens, demonstrating that formation of inside-out enteroids from isolated villi in floating culture extends across at least two avian species.

We then expanded the range of species analysed to explore the phenotypic plasticity of mammalian intestinal crypts in a liquid environment. Mouse crypts (Fig. 2m) seeded in Matrigel predictably developed into enteroids with an internally polarised lumen, forming spheroid structures by 2 days of culture (Fig. 2n) and multiple budding structures by 7 days. In contrast, isolated murine crypts cultured in Mouse IntestiCult medium alone failed to maintain structural integrity and at 2 days of culture no enteroid-like structures were visible (Fig. 2o).

### Floating chicken enteroids reproduce the cellular diversity of the intestinal epithelium in vivo

**Fig. 3:**
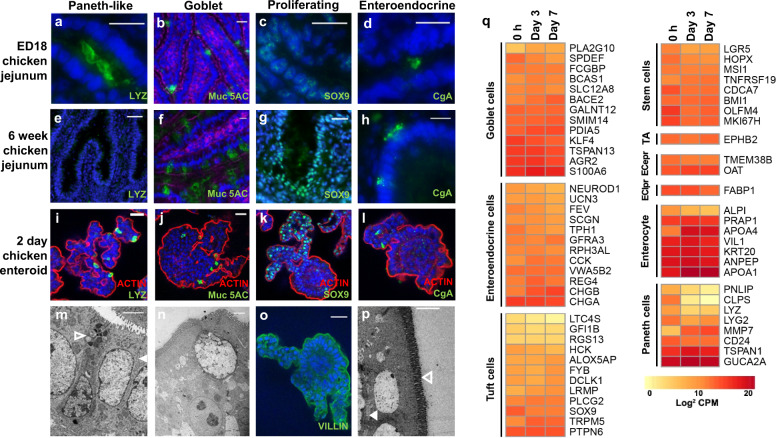
Multicellular composition of chicken enteroids. Confocal images of **a**–**d** embryonic jejunum, **e**–**h** 6 week old chicken jejunum, and **i**–**l**, **o** embryonic chicken enteroids at 2 days of culture. All cells are counterstained with DAPI (blue). The cells are stained for **a**, **e**, **i** Lysozyme C (green, Paneth cells), **b**, **f**, **j** Muc5AC (green, goblet cells), **c**, **g**, **k** SOX9 (green, proliferating cells) and **d**, **h**, **l** Chromogranin A (green, enteroendocrine cells). **i**–**l** Chicken enteroids stained to detect F-actin-expressing brush border (red). **m** Transmission electron microscopy of chicken enteroids (4 h in culture) demonstrates an enterocyte (closed arrow) and Paneth cell (open arrow). **n** TEM of chicken enteroids (7 days in culture) demonstrates a goblet cell. **o** Confocal image of chicken enteroid stained for villin (green, epithelial microvilli). **p** TEM of chicken enteroid 7 days in culture, enterocyte basal lamina (closed arrow) and microvilli (open arrow). Scale bar: **a**–**l**, **o** 20 µm. **m**, **n**, **p** 2 µm. Images **a**–**p** are representative of data from at least 3 independent cultures each containing 2–3 embryos. **q** Expression of intestinal epithelial cell lineage-specific genes in freshly isolated villi (0 h) and enteroids at 3 and 7 days of culture compared by RNA sequencing analysis. Heat maps show the relative expression levels (log2 counts per million reads) of a range of mammalian epithelial cell lineage-related genes. TA: transit amplifying cells, ECepr: early enterocyte precursor cells, EClpr: late enterocyte precursor cells. RNA sequencing data is representative of 3 independent experiments, each comprising of 2 technical replicates, each containing 3 embryos.

To investigate whether the multilobulated floating chicken enteroids displayed the array of cell types characteristically present in the gut epithelium in vivo, immunofluorescent staining and TEM were performed. Enteroids from villus isolation to 7 days of culture were compared with embryonic and immunologically mature chicken jejunal tissue sections. In human and mouse small intestinal epithelium, lysozyme C is synthesized and secreted by crypt dwelling Paneth cells^25^, whereas in the embryonic chicken small intestine and floating chicken enteroids, lysozyme C-expressing epithelial cells were observed scattered throughout the epithelium (Fig. 3a, i; TEM Fig. 3m). Assuming chicken Paneth-like cells play a similar stem-cell supportive role as their murine counterparts, this staining pattern could also reflect the multiple sites of proliferation expected along the villi in late stage embryos and newly hatched chicks^26,27^. Lysozyme C was not detected by immunohistochemistry in our mature chicken gut sections (Fig. 3e). This could reflect a reduction in expression with age^28^, or as a more recent publication has reported Lysozyme C mRNA expression up to 8 weeks post-hatch, reduction of protein expression through complex gene regulatory mechanisms^29^. SOX9, which is expressed by stem cells, transit-amplifying cells and terminally differentiated Paneth cells, was concentrated in epithelial cells lining the embryonic villi and mature chicken crypts (Fig. 3c, g). Localisation of both SOX9 and EdU (Fig. 1f) to the embryonic enteroid bud epithelium (Fig. 3k) indicates these villus-like structures are sites of proliferation and differentiation of intestinal stem and progenitor cells. Goblet cells (Muc5AC^+^ cells, Fig. 3b, f, j; TEM Fig. 3n) and enteroendocrine cells (chromogranin A^+^ cells, Fig. 3d, h, l) were scattered throughout the enteroids, and in embryonic and mature chicken gut sections. Uniformly polarized enterocytes with clear apical villin-expressing brush borders (Fig. 3o), external microvilli (TEM, Fig. 3p open arrow) and internal basal lamina (Fig. 3p closed arrow) lined the enteroid epithelial surface. Globally the distribution patterns of the cell types in the enteroids appeared to be similar to that observed in the embryonic intestine in vivo.

The transcriptional profile of embryonic enteroids at 0, 3 and 7 days post cultivation suggested expression of gene sets characteristically associated with mammalian Paneth cells, enterocytes, goblet cells and enteroendocrine cells (Fig. 3q)^24^. In addition, expression of classical markers for cell subpopulations that could not be detected by microscopy, including stem cells, transit amplifying cells and tuft cells, were identified^4,24,30^. The expression of these genes for most cell-types was relatively stable during the cultivation period, suggesting that the enteroids accurately recapitulate the cellular diversity of the in vivo epithelium for at least 7 days. However, particularly within the Paneth cell and enterocyte gene-sets, there were some markers with striking transcript changes. Across the time-points there was upregulation of the Paneth cell-associated gene *MMP7* which activates antimicrobial peptides. Conversely there was reduced expression of the lipase related genes *CLPS* and *PNLIP* which may be a consequence of the adaptation to the culture medium. In the enterocytes, *ALPI* (encoding alkaline phosphatase) was slightly downregulated over time, whilst other classical markers e.g. *VIL1, ANPEP, KRT20* remained stable. This may be due to species-specific differences between avian and murine enterocytes or a consequence of their adaption to in vitro culture media since alkaline phosphatase expression can be regulated by dietary macronutrients and fasting (reviewed^31^). Across the cultures there was an upregulation of many lipid digestion-related genes that map to the PPAR signalling pathway, a key regulator of intestinal metabolism. This included the enterocyte marker *APOA1* as well as *APOA5, HMGCS2, PLTP, ME1* and *CYP8B1*. There was also increased expression of enterocyte-related marker *APOA4* which is involved in lipoprotein metabolism, brush border enzymes *LCT* and *SLC9A3R1*, the main apical sodium-dependent enterocyte transporter *SLC13A1*, as well as *ENPEP* and *ACE2* which participate in control of glucose uptake, sodium and water absorption, and digestion and absorption of peptides at the brush border. Although it is unclear whether the described transcript changes are functionally significant, that the chicken enteroids develop strong expression of digestion-related genes and associated pathways indicates that the in vitro conditions contain the cues for maturation to a post-hatch gut model. This hardwiring of the developmental program into the fetal gut epithelium has previously been noted in murine fetal enteroids which retain fetal properties for a limited time before continuing to develop into adult-like enteroids^32^.

**Fig. 4:**
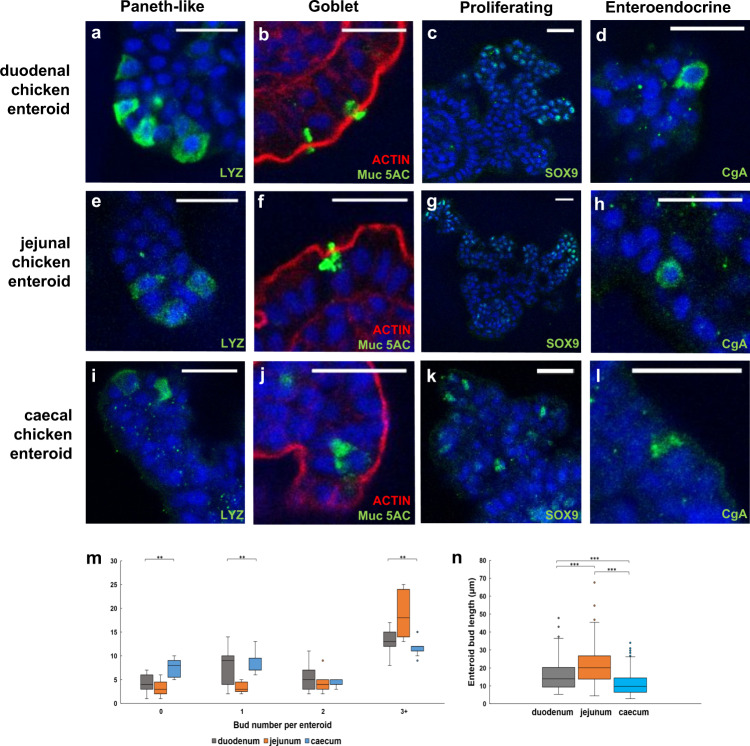
Site-specific chicken enteroids demonstrate in vivo multicellular composition and architecture. **a**–**l** Confocal images of embryonic chicken enteroids at 2 days of culture grown from the duodenum, jejunum and caeca. Stained for **a**, **e**, **i** Lysozyme C (green, Paneth cells), **b**, **f**, **j** Muc5AC (green, goblet cells), **c**, **g**, **k** Sox9 (green, proliferating cells), and **d**, **h**, **l** Chromogranin A (green, enteroendocrine cells). All counterstained with DAPI (blue). **b**, **f**, **j** stained to detect F-actin-expressing brush border (red). Scale bar: 20 µm. Images are representative of data from at least 3 independent cultures, each containing 2–3 embryos. **m** Enteroids were cultured for 2 days and the number of villus-crypt like structures or buds, i.e. 0, 1, 2, 3 or more per enteroid, were counted. After 2 days of culture there were statistically significant differences in numbers of buds between the different regions of the small intestine. The caecal enteroids more often lack buds (0) or only have 1 bud compared to duodenal and jejunal enteroids, whereas jejunal enteroids have more buds (3+) in comparison to duodenal and caecal enteroids. **n** The length of the buds resembled the in vivo architecture with jejunal and duodenal buds measuring significantly longer than caecal buds. Box and whisker plot data derived from 3 independent experiments each containing 2–3 embryos, at least 260 enteroids and 150 buds measured per location for **m** and **n**, respectively. Statistics performed using a Kruskal–Wallis test; **m** 0 budding, **p 0.001, H = 14.06, df = 2; 1 budding, **p 0.001, H = 13.42, df = 2; and more than 3 budding, **p 0.001, H = 13.62, df = 2, enteroids between the duodenal, jejunal and caecal cultures. **n** ***p 0.0001, H = 110.08, df = 2, with *post hoc* Mann–Whitney *U*-tests (two-sided); duodenum–jejunum, ***p 0.0001, *W* = 23230, 95% CI for difference (−6.820, −3.266); duodenum–caecum, ***p 0.0001, W = 29697.5, 95% CI for difference (13.914, 9.780); jejunum–caecum, ***p 0.0001, *W* = 46327.5, 95% CI for difference (7.249, 10.707).

In order to provide site-specific models for in vitro infection studies, differentiated chicken duodenal, jejunal and caecal enteroids were individually prepared (Fig. 4a–l). The caecal enteroids utilised the same growth requirements as small intestinal enteroids. Characterisation of these enteroids showed they contained a similar abundance of cell types, as well as an inside-out conformation. After 2 days of culture there were statistically significant differences in the numbers of buds between the different regions of the small intestine (Fig. 4m). The caecal enteroids more often lack buds (0) or only have 1 bud compared to duodenal and jejunal enteroids. The jejunal enteroids have more buds (3+) in comparison to duodenal and caecal enteroids. In addition, the length of the villus-crypt structures or buds resembled the in vivo architecture, as the buds differentiated from jejunal and duodenal tissue were significantly longer than those from caecal enteroids (Fig. 4n).

### Epithelial barrier integrity and cell stress

**Fig. 5:**
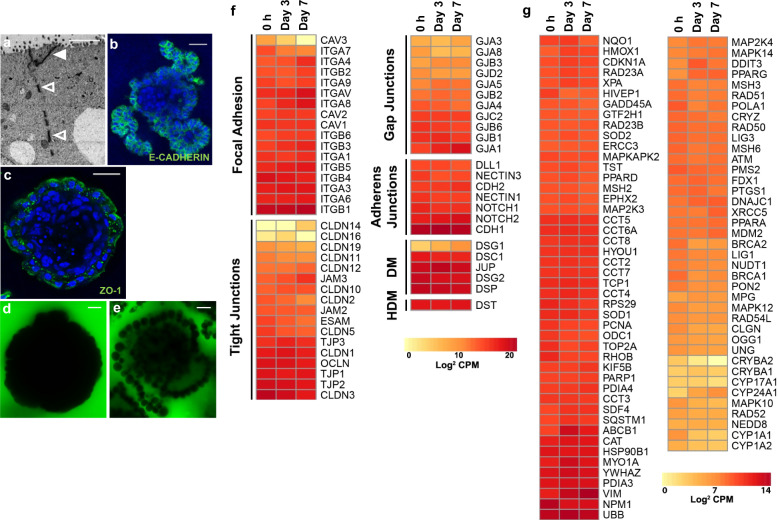
Chicken enteroids display epithelial barrier integrity, express cell junction related genes and minimally alter stress-related genes. **a** Transmission electron microscopy of a chicken enteroid (7 days of culture) demonstrates tight junctions (closed arrow) and desmosomes (open arrows). **b**–**c** Confocal images of chicken enteroids (2 days of culture) stained for **b** E-cadherin (green, adherens junctions) and **c** ZO-1 (green, tight junctions) and counterstained with DAPI (blue). **d**–**e** Confocal images of chicken enteroids (2 days of culture) immersed in FITC-dextran 4 kDa showing epithelial barrier integrity in **d** untreated and **e** loss of barrier integrity after EDTA-treatment. Scale bar: **a** 2 µm, **b**–**e** 20 µm. Images are representative of data from at least 3 independent cultures each containing 2–3 embryos. **f**–**g** Expression of **f** epithelial cell junction-related genes and **g** cell stress-related genes in freshly isolated villi (0 h) and chicken enteroids at 3 and 7 days of culture was compared by RNA sequencing analysis. **f** Heat maps show the expression levels (log2 counts per million reads) of a range of epithelial cell junction-related genes. DM: desmosomes HD: hemi-desmosomes. **g** Heat map shows minimal change in expression levels of a range of mammalian cell stress-related genes over 7 days of culture. RNA sequencing data is representative of 3 independent experiments, comprising 2 technical replicates, each containing 3 embryos.

The innermost layer of the intestinal luminal surface consists of a single cell thick epithelial lining which acts as a barrier, preventing the entry of harmful molecules and microbes while still allowing the selective passage of dietary nutrients, ions, and water^33^. Tight junction proteins together with adherens junctions and desmosomes are essential gut epithelia barrier components which maintain physiological homeostasis^34^. By immunostaining for two major cell adhesion molecules and using TEM, we demonstrated the presence of these junctions in chicken enteroids. Desmosomes and tight junctions (Fig. 5a) were visualised with TEM, adherens junctions were identified by intercellular E-cadherin expression (Fig. 5b) and the tight junction-associated protein ZO-1 was expressed in the epithelial layer (Fig. 5c).

In order to determine whether the cell-cell junctions were functional, the enteroids were immersed in 4 kDa FITC-dextran. Enteroids treated with 5 mM EDTA, which disrupts tight junctions, were used as a positive control. This analysis showed that untreated enteroids excluded the FITC-dextran, demonstrating mechanical integrity through intact intercellular junctions (Fig. 5d). The EDTA-treated enteroids, in contrast, allowed permeation of FITC-dextran through the intercellular spaces, indicating breakdown of the epithelial barrier (Fig. 5e).

Transcriptional analysis demonstrated that the enteroids expressed a large range of genes encoding components of mammalian focal adhesions, tight junctions, gap junctions, adherens junctions and desmosomes (Fig. 5f). Expression of these genes was generally stable throughout the culture period. In addition, although a range of cell stress associated genes (derived from murine studies^1^) were expressed in the enteroids, there was no significant evidence of modulation of their expression across the time points (Fig. 5g). Steady expression of both gene sets throughout the time-points is indicative of stable enteroid cultures over 7 days.

### Immune cell component

**Fig. 6:**
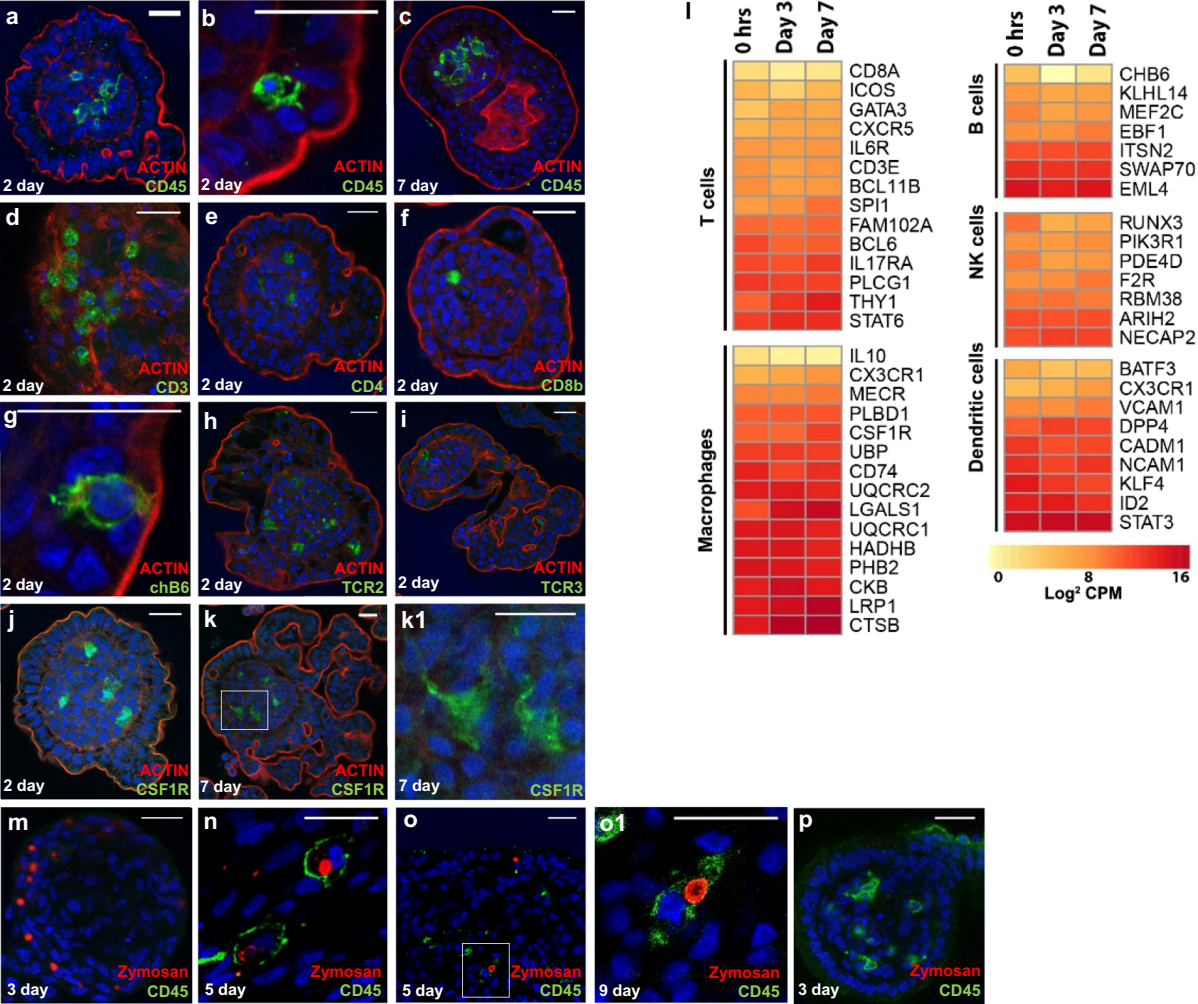
Composition and functionality of immune cells within chicken enteroids. **a**–**i** Confocal images of chicken enteroids stained for leukocyte markers (green) at 2 or 7 days of culture. All enteroids are counterstained with DAPI (blue) and Phalloidin (red). **a**–**c** Enteroids stained for CD45 (green) showing leukocytes in the **a**, **c** lamina propria and **b** epithelium. Enteroids at 2 days of culture stained for **d** CD3, **e** CD4, **f** CD8β, **g** chB6, **h** TCR2 (chicken αβ_1_ TCR), **i** and TCR3 (chicken αβ_2_ TCR). **j**–**k** Enteroids cultured from *CSF1R*-eGFP transgenic embryos at **j** day 2 and (**k1** magnification of **k**) day 7 of culture. Scale bar: 20 µm. **l** Expression of immune cell-related genes in freshly isolated villi (0 h), 3 day and 7 day chicken enteroids was compared by RNA sequencing analysis. Heat maps show the expression levels (log2 counts per million reads) of a range of immune cell-related genes. RNA sequencing data is representative of 3 independent experiments, each comprising of 2 technical replicates, each containing 3 embryos. **m**–**p** pHrodo zymosan bioparticles (red) were added to 3 day enteroid cultures and visualised **m** in the epithelium after 8 h and **n** in CD45^+^ leukocytes (green) after 48 h. (**o1** magnification of **o**) Zymosan was also added to 7 day enteroid cultures and phagocytosis of zymosan by leukocytes in the ‘lamina propria’ core was visualised after 48 h. **p** Zymosan uptake was blocked in 3 day enteroid cultures treated with Cytochalasin D, which inhibits actin polymerization. Scale bar: 20 µm. Images are representative of data from at least 3 independent cultures each containing 2–3 embryos.

Since embryonic enteroids develop from intestinal villi we determined whether they also contained immune cells derived from the intestinal lamina propria. Using immunohistochemistry we identified CD45^+^ leukocytes scattered throughout the central cell-dense core of the enteroids (Fig. 6a–c) and occasionally within the epithelium (Fig. 6b) at 2 days (Fig. 6a, b) and 7 days (Fig. 6c) of culture. Subsequent immunostaining reflected the presence of cytoplasmic CD3^+^ cells, typical of NK cells which have been detected in embryos from ED14^35^, CD4^+^ cells and CD8β^+^ cells (Fig. 6d–f). ChB6^+^ cells were identified which are indicative of both B cells and NK cells (Fig. 6g)^36^. Occasional chicken αβ_1_ TCR^+^ (TCR2) and chicken αβ_2_ TCR^+^ (TCR3) cells were found scattered through the enteroid lamina propria core (Fig. 6h–i), appearing in the embryonic intestine a couple of days earlier than previous studies have reported^37,38^. This could reflect breed-specific variation or changes to laying stock in the intervening 30 years of genetic selection. Embryonic chicken intestines cultured from *CSF1R*-reporter transgenic chicken embryos, which express eGFP in cells of the myeloid lineage, were used to visualise tissue mononuclear phagocytes^39^. Imaging of *CSF1R*-eGFP transgenic chicken enteroids showed the presence of multiple *CSF1R* transgene^+^ cells, indicating the presence of macrophages and dendritic cells within the enteroid core at 2 days (Fig. 6j) and 7 days (Fig. 6k, k1) of culture.

Further transcriptional analysis of mRNA from floating enteroids showed the expression of gene sets encoding various leukocytes of the mammalian enteric immune system (Fig. 6l), with relatively stable expression across the 7 days of culture. Our analyses confirmed strong expression of macrophage-related genes *CSF1R, CTSB, LRP1, CKB, UQCRC1, PHB2* and *HADHB*. Gene-sets associated with NK cells, T cells, dendritic cells, and B cells were also represented, but their expression profiles suggest they are present in lower numbers than macrophages.

To demonstrate the functional ability of the immune cell populations to phagocytose particles from the surrounding medium, we added pHrodo zymosan bioparticles to 3 and 7 day enteroid cultures. The enteroids were imaged 8 and 48 h after the addition of zymosan. The pH-sensitive zymosan bioparticles fluoresce red in acidic pH (phagosomes), and their presence could be detected within epithelial cells after 8 h (Fig. 6m) and within CD45 + leukocytes in the enteroid core after 48 h (Fig. 6n–o1). Phagocytosis by leukocytes in the lamina propria was also detected 48 h after adding zymosan to 7 day enteroid cultures. This indicates that the leukocytes were still functionally active after 7 day of culture. The uptake of the pHrodo zymosan bioparticles required active phagocytosis as this activity was blocked when the enteroids were treated with cytochalasin D (Fig. 6p).

### Propagation

**Fig. 7:**
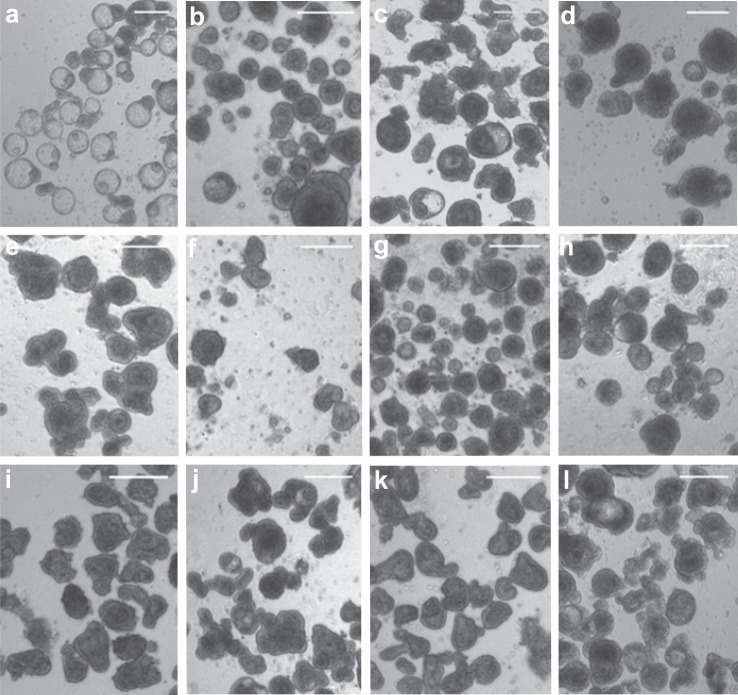
Chicken enteroid propagation. **a** Brightfield images of representative enteroid cultures supplemented with EGF, R-spondin and Noggin at day 1 and **b** day 9 compared to FOM-only at **c** day 1 and **d** day 9 of culture. Chicken enteroids at **e** day 4 culture, **f** immediately post-passage and **g** day 3 post-passage in plain and **h** growth factor supplemented media. Cryopreserved crypts at **i** point of thaw and **j** after 4 days of culture compared to **k** freshly isolated crypts then **l** cultured for 4 days. Images **a**–**i** are representative of at least 3 independent cultures each containing 2–3 embryos. Scale bar: 200 µm.

Supplementation of floating organoid media (FOM) with human growth factors Noggin, R-spondin and EGF, all typically essential for mammalian enteroid growth^1^, conferred no obvious advantage in development or resultant architecture of embryonic floating enteroids during the first 9 days of culture (Fig. 7a–d). The growth factors did initially support large numbers of empty enterosphere-like structures with budding events (Fig. 7a) which persisted for a couple of days. However, subsequently the cultures began to resemble those without growth factor supplementation where the enteroids have solid spheroid cores (Fig. 7b, d).

To attempt to passage the 7 day old enteroids (Fig. 7e) we used mechanical disruption to remove the crypt-domain buds (Fig. 7f). Despite the addition of supplements reported to improve passage in other species (Y-27632, LY2157632, SB202190, EGF, R-spondin and Noggin)^2,4,40^, there was no post-passage growth or budding of the enteroids after 3 days in either un-supplemented (Fig. 7g) or supplemented FOM (Fig. 7h).

However, successful recovery of embryonic small intestinal villi from cryopreserved stocks (Fig. 7i) was demonstrated by their subsequent resuscitation and growth into extensively budding enteroids (Fig. 7j) at similar rates to freshly isolated villi (Fig. 7k,l). Two day old enteroids were also successfully recovered from cryopreserved stocks (supplementary Fig. 3).

### Chicken enteroids are susceptible to infection by bacteria, eukaryotic parasites and viruses

**Fig. 8:**
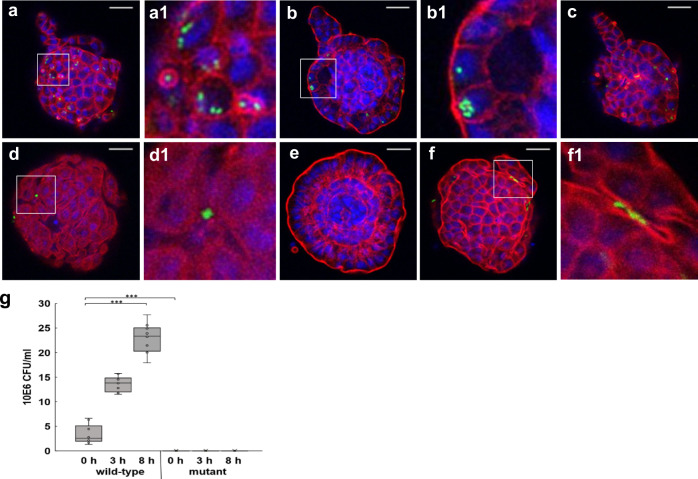
Chicken enteroids as a model for host–bacterial interactions. **a**–**f** Representative z-axis projections of chicken enteroids 2 days in culture whole-mount stained to detect cell nuclei (DAPI, blue) and F-actin-expressing brush border (red). Enteroids incubated with **a**–**c** wild type *S*. Typhimurium-GFP (green) and **d**–**f** mutant non-invasive *S*.Typimurium-GFP (green) at 4 h. Magnified images of **a1** actin remodelling, **b1** intracellular bacteria and **d1**, **f1** lack of actin remodelling and intracellular bacteria. Images are representative of data from at least 3 independent cultures each containing 2–3 embryos. Scale bar: 20 µm. **g** Bacterial net replication assay confirmed *Salmonella* counts were significantly increased for enteroids infected with wild-type versus mutant *Salmonella* strains. Box and whisker plot represent bacterial count from ~800 infected enteroids post high-dose gentamicin treatment. Data derived from 5 independent experiments with 2–3 embryos per culture. ***p < 0.0002, *W* = 55, 95.5% CI for n1–n2 is (−419850, −189760) using a Mann–Whitney *U*-test (two-sided). The assay also showed wild-type *Salmonella* replicated in the enteroids over 0–8 h. ***p < 0.0001, *R*^2^ = 0.73, df = 29 using a linear regression test.

Using a range of important zoonotic and avian-specific pathogens we next determined whether the inside-out phenotype facilitated uncomplicated infection studies by simply adding these microorganisms to the media. Enteroids were incubated for 4 h with either a wild-type *S*. Typhimurium strain or a non-invasive mutant strain, defective in the *Salmonella* pathogenicity island 1 (SPI1)-encoded T3SS. *S*. Typhimurium uses effector proteins translocated by the SPI1 T3SS to induce host-cell actin remodelling on the apical surface of polarized epithelial cells. These membrane ruffles are a well-characterised feature of *Salmonella* virulence, promoting internalization of the pathogen by non-phagocytic cells (reviewed in ref. ^41^). After 30 min incubation of the enteroids with wild-type *S*. Typhimurium, the bacteria were visualised in contact with the apical epithelial surface. After 4 h of infection, dense actin rings surrounded individual bacteria (Fig. 8a1) and large numbers of bacteria were disseminated intracellularly throughout the enteroids (Fig. 8b1). In contrast, few non-invasive mutant *S*. Typhimurium were found in contact with the microvilli, no dense cytoskeletal modifications were visualised (Fig. 8d1, f1) and only occasional intracellular bacteria were identified after 4 h of infection. Bacterial net replication assays confirmed significantly increased numbers of wild-type *Salmonella* in enteroids after 1 h of infection, compared to those exposed to the mutant strain, and only the enteroid cultures infected with wild-type strain demonstrated significant net replication (Fig. 8g).

**Fig. 9:**
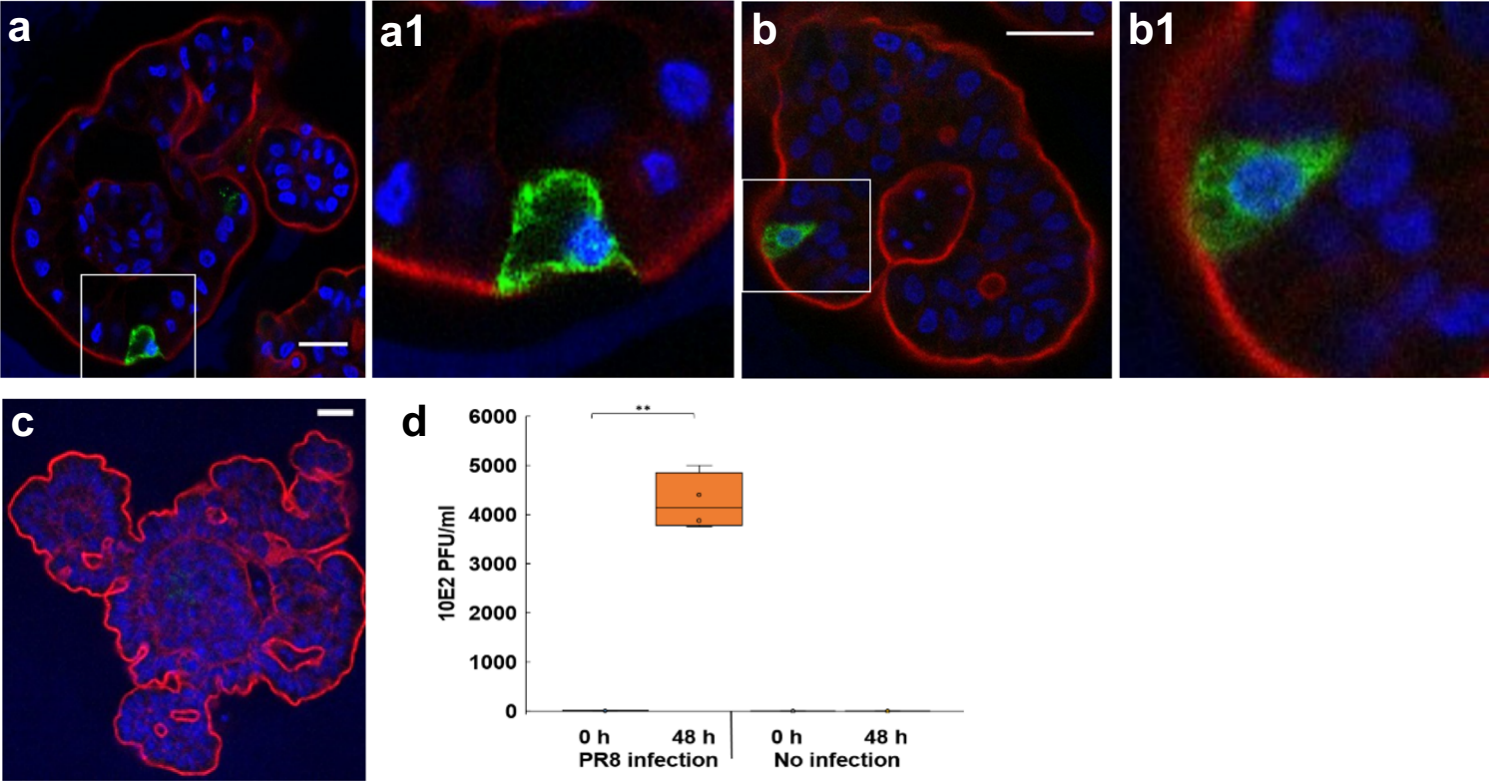
Chicken enteroids as a model for host–viral interactions. **a**–**b** Representative z-axis projections of chicken enteroid 3 days in culture whole-mount stained to detect cell nuclei (DAPI, blue), F-actin-expressing brush border (red) and virus nucleoprotein (green) after incubation with influenza A virus (PR8) for 48 h. **a1** Magnified image of **a**. **b2** Magnified image of **b**. **c** Isotype control. Images are representative of data from at least 3 independent cultures each, containing 2–3 embryos. Scale bar: 20 µm. **d** Box and whisker plot represent viral titers, determined by plaque assay in supernatant from ~800 infected enteroids at 0 and 48 h post-wash. Data is derived from 4 independent experiments each containing 2–3 embryos and ~800 seeded enteroids/well. **p < 0.001, *T* = −14.98, 95% CI for mean difference (−514720, 334344), df = 3 using a paired *t*-test (two-sided).

Next, the enteroids were incubated with influenza A virus strain PR8 (H1N1) for 1 h, washed and then cultured for a further 48 h. Expression of viral nucleoprotein (NP) was detected within the epithelium of the enteroids after 48 h of infection (Fig. 9). Viral replication in the enteroids was verified by measuring infectious virus titers in supernatant of infected enteroids, by plaque titration on MDCK cells. Titers significantly increased from 0 to 48 h post-wash compared to mock infected controls (Fig. 9d).

**Fig. 10:**
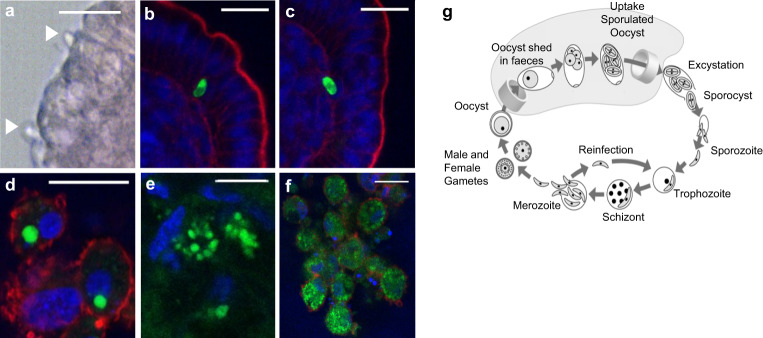
Chicken enteroids as a model for host–protozoal interactions. **a** Brightfield image of sporozoites (closed arrow) entering caecal enteroid at 1 dpi. **b**–**f** Representative z-axis projections of chicken caecal enteroids whole-mount stained to detect cell nuclei (DAPI, blue), F-actin-expressing brush border (red) after incubation with PKH-67 labelled *E. tenella* (green). **b** Sporozoite at 2 dpi within enteroid epithelial cell and **c** migrating through basement membrane into lamina propria. **d** *E. tenella* trophozoite-like structures. **e**–**f** Schizogeny within enteroid epithelial cell at 9 dpi. **g** Schematic diagram of *E. tenella* lifecycle, source: impextraco.com. Scale bar: **a**–**d**, **f** 20 µm, **e** 10 µm. Images are representative of data from 2 independent experiments each with 2–3 technical replicates containing >3 embryos.

The apicomplexan protozoa of the genus *Eimeria* are one of the major parasitic diseases of poultry. Following oral infection in vivo, the *E. tenella* sporozoites enter exclusively the caecal epithelial cells, migrate through the lamina propria to undergo multiple rounds of asexual multiplication at the base of the crypts, before eventually undergoing sexual multiplication. Since each developmental stage of *Eimeria* harbours a distinct number of parasitic divisions, we used a combination of brightfield and fluorescence microscopy to determine whether the enteroid cultures could support parasite replication. In order to visualise the parasites, *E. tenella* sporozoites were stained with a fluorescent cell-membrane tracking dye, PKH67. At 1 day post-infection (dpi) sporozoites were identified in contact with the enteroid apical epithelial surface (Fig. 10a) and by 2 dpi they were observed inside enteroid cells (Fig. 10b, c). *E. tenella* subsequently divided within the enteroid cells (Fig. 10d–f), as determined by the size and increased number of PKH67 + parasites within a singular cell. The fluorescent membrane divisions in the enteroid cells correlated with what would be expected for distinct parasite lifecycle stages (Fig. 10g). To confirm the sexual replication stage was reached, expression of *EtGAM56*, which encodes a macrogamete specific protein incorporated into the oocyst wall, was demonstrated in *E. tenella* infected enteroids at 5, 7, and 9 dpi, but not at 2 dpi (supplementary Fig. 4).

## Discussion

Mammalian 3D enteroids already facilitate research into multicellular biological mechanisms, proving to be more physiologically relevant than traditional monolayer cultures of cell-lines and limiting the need for in vivo studies (reviewed in ref. ^42^). The use of established protocols originally devised for the culture of murine crypts has so far failed to generate avian enteroids that are representative of the in vivo intestine, demonstrating few if any villus- and crypt-like structures and differentiated cells^19–21^. Here we describe a method to successfully differentiate self-organising, extensively budding avian enteroids from both embryonic and mature chicken gut that mimic the in vivo architecture and epithelial characteristics of the avian intestine without the use of a gel scaffold. Two striking features of avian enteroids grown floating in culture are, (1) their inside-out conformation, with the apical brush border facing the media, and (2) the innate presence of leukocytes, creating a valuable, natural epithelial-leukocyte co-culture enteroid model.

The isolation of villi instead of crypts, which are employed in mammalian enteroid cultures, from embryonic birds is because the crypt structures are only at a rudimentary stage of development. It takes till 48 h post-hatch for intestinal epithelial invaginations to complete with crypt enlargement and fission continuing over 9 days post-hatch^27,43^. Budding enteroids can be developed from villus tips in the embryonic chicken because almost all villus cells in poultry are proliferative at hatch with cell mitosis playing an important role in post-hatch hyperplasia^27^. The enteroid transcriptome indicated the embryonic cultures continue development into a post-hatch intestinal model. This is also reflected by their growth characteristics which mirror in vivo intestinal post-hatch development with intensive cryptogenesis and villus proliferation in the first 24 h of culture^27^. Crypts from mature chickens, cultured in a manner similar to embryonic villi, also formed multilobulated enteroids providing evidence that enteroid culture techniques can be successfully applied to other ages of bird. However, enteroids prepared from older birds have poorer budding and longevity as compared to those derived from embryonic individuals which, in part, is likely due to a decrease in the proportion of proliferative cells, stem cells, lining the intestinal epithelium with age^27^.

Seeding chicken crypts or villi in Matrigel, a biologically active basement membrane matrix, resulted in minimally budding spheroid structures, which polarised like their mammalian gel-embedded counterparts with internal microvilli. These hollow structures closely resemble murine foetal spheroids, which demonstrate Wnt-dependent indefinite self-renewing properties but display a poorly differentiated phenotype^32^. This is in stark contrast to the extensively budding, differentiated, inside-out enteroids that are produced when chicken villi are floated in suspension. In 1990 Madin-Darby canine kidney epithelial spheroids were noted to have a polarity where the basal cell surface faced out when in collagen gel, but the apical surface faced out when in suspension^44^. A recent publication by Co et al.^45^ repeated this phenomenon with human and murine enteroids^45^. These mammalian cultures were initially established as traditional gel-embedded enteroids for 7–20 days, before being moved into suspension where their epithelial cell polarity reversed after 3 days. ECM proteins have been identified as one of the key regulators of this epithelial polarity change, specifically through β1 integrin signalling^45–47^. A scaffold-free system for poultry enteroids was previously investigated by Acharya et al.^22^ but, in sharp contrast to our system, only produced spheroid structures without defined crypt- and villus-like domains, similar to the chicken enteroids grown in environments otherwise successful for mammals^22^. Other mechanisms that may contribute to the differences in cell budding, differentiation and polarity in these chicken enteroids include isolation techniques, ligand-receptor interactions with soluble growth factors^48^ as well as the mechanical properties of Matrigel^49–51^.

The rapid dissociation of floating murine crypts into disorganised clumps of cells is likely due to loss of integrin signalling through basal membrane attachments. Previous studies have shown this induces rapid anoikis in intestinal epithelial cells^52^. Several mammalian epithelial cell types however, have been able to overcome cell-matrix-anchorage associated anoikis when cell-cell-contact is preserved, likely through E-cadherin’s compensatory mechanisms^53^. Species differences in cell adhesion protein dynamics may explain why loss of cell-matrix-anchorage does not appear to negatively affect avian crypts. Other contributing factors may include differences in polarity-reversal rates, crypt isolation protocols, structural integrity of isolated crypt/villi and soluble growth factors.

Floating chicken enteroids have demonstrated viability and functionality for at least 9 days of culture. Proliferation of the epithelium and cells in the inner core, increase in digestion-related gene expression and phagocytosis of zymosan by lamina propria leukocytes indicates this complex intestinal model sustains in vivo functions for over a week. Without the ability to passage, these enteroids do not have the longevity of enteroids grown in more conventional culture systems^1,2^. This is likely due to the relative absence of exogenous growth and/or inhibitory factors. However, in the study by Co et al. (2019)^45^ the floating mammalian enteroids can only be maintained for 5 days in culture and evidence for long-term propagation was not reported. Therefore, the influence of the mechanical environment and epithelial polarity may also play a role. Since the avian enteroids mature more quickly than their mammalian counterparts and can be used after 24–48 h for further experimentation, the lack of long-term culture longevity is not a substantially prohibitive factor. In addition, successful biobanking of large amounts of enteroid-forming embryonic villi tips and 2 day old enteroids through cryopreservation enables the use of the same material for multiple studies and by other labs. This helps to address the 3Rs as it removes the need to cull birds for every experiment.

The need for a species-specific intestinal epithelial-immune cell model is a necessary advancement for studying coordinated enteric immune responses in avian health and disease. Identification of both intra- and sub-epithelial leukocytes in our enteroids show we have cultivated such a model for the chicken, by much simpler means than the staged murine and human co-culture protocols previously published^8,9,54^. Accurately mapping the location of CSF1R^+^ cells using whole-mount enteroids derived from *CSF1R*-eGFP transgenic chickens, and evidencing leukocyte functionality in the lamina propria core using phagocytosis studies demonstrates the versatility of this in vitro tool. Macrophages have also been shown to improve intestinal epithelial cell maturation and barrier stability therefore their presence may in part explain why chicken enteroids require no external growth factors during the first week of culture^8^. The application of single cell sequencing with the enteroids would be beneficial to further explore the stage of maturation and function of both immune and epithelial cell types, and to fully characterise the genes expressed by individual cell populations present.

The inside-out polarity of avian enteroids allows for uncomplicated imitation of the natural infection process by simply adding microorganisms to the same media as the enteroids, as demonstrated in this study for bacterial, viral and protozoal pathogens. Actin remodelled ruffles were evident on the enteroid epithelial surface after wild-type but not non-invasive mutant *S*. Typhimurium infections. To our knowledge, this is the first time *Salmonella*-induced ruffles have been demonstrated in polarised primary avian intestinal epithelial cells. The difference in brush border interactions, as well as stark differences in microbial invasion and replication between these *Salmonella* strains, show the enteroids mimic in vivo intestinal cellular machinery making them promising experimental platforms to investigate *Salmonella* infections.

Both low and highly pathogenic avian influenza have been recovered from the intestine of infected chickens^55^. Other enterotropic viruses in poultry that cause significant losses to the industry, are difficult to research in vivo and few specific treatments exist^56–58^. By demonstrating successful invasion and replication of influenza A virus in the enteroids, we have confirmed they are capable host-models to recreate viral infection of the avian intestinal mucosa. Indeed, although chickens aren’t considered to be susceptible to SARS-CoV-2 coronavirus^59^, the *ACE2* expressing chicken enteroids could be used to determine whether the avian gut epithelium has the potential to replicate the virus, as recently demonstrated using human enteroids^14^.

*Eimeria* belong to the phylum Apicomplexa, a large group of intracellular parasites containing some of the most widespread protozoa of humans and animals including *Neospora*, *Toxoplasma* and *Cryptosporidium* species. The lifecycle of *E. tenella* has only been partially replicated in vitro, most successfully to date in primary chicken kidney cells and a chicken lung cell line (CLEC-213)^60,61^. However, in vivo the parasite’s sporozoites are exclusively trophic to the chicken caecum. In a chicken caecal model, we show *E. tenella* sporozoites infecting the epithelium and subsequently undergoing both schizogeny and gametogeny, as verified by measuring macrogamete gene expression. In order to identify the infection efficiency of site-specific parasites in site-specific enteroids, further comprehensive studies are required.

Overall, we have successfully optimised a protocol to prepare differentiated leukocyte-containing avian enteroids with an accessible epithelial layer. These enteroids reflect the 3D architecture, polarity, barrier function, and cellular composition of their in vivo counterparts qualifying them as an effective in vitro model of the post-hatch and mature chicken intestine. Moreover, the enteroids derived from different parts of the small and large intestine resemble the in vivo architecture, i.e. length and number of villus-crypt structures and the cellular composition. The chicken enteroids have been successfully infected with *S*. Typhimurium, influenza A virus A and *E. tenella* evidencing their flexible use in the study of infections with pathogenic microorganisms of importance to animal and human health. The presence of both epithelial and immune cell types means the enteroids closely recapitulate the cellular diversity and functionality of the chicken intestine, and can be successfully used to interrogate immune responses to enteric pathogens. The inside-out phenotype and leukocyte component of these enteroids provides an inexpensive and uncomplicated model in which to research host–pathogen interactions, and their scope could easily be extended for pharmaceutical, nutritional and developmental studies.

## Methods

### Animals

Experiments were performed using ED18 to 9 week old Hy-Line Brown chickens (*Gallus gallus*), ED17 *CSF1R*-eGFP transgenic chickens^39^ and 2 day old quail (*Coturnix coturnix*) obtained from the National Avian Research Facility, Edinburgh, UK. Five month old C57BL/6 mice were provided by the Biological Research Facility, University of Edinburgh, UK.

Ethical approvals were obtained from The Roslin Institute’s and University of Edinburgh’s Animal Welfare Ethics Review Board. The experiments were also performed under the authority of UK Home Office Project Licences (PA75389E7, PE263A4FA) in accordance within the guidelines and regulations of the UK Home Office ‘Animals (scientific procedures) Act 1986.

### Isolation of avian intestinal tissue containing stem cells

The small intestine was removed post-mortem, cut open longitudinally then into 5 mm sections and collected into Ca^2+^- and Mg^2+^-free Phosphate-buffered saline (PBS) and washed. The tissue was digested in Dulbecco’s Modified Eagle’s Medium (DMEM) (Thermo Fisher Scientific (TFS)) with 0.2 mg/mL Collagenase from *Clostridium histolyticum* Type IA (Merck) at 37 °C for 50 min. The tube was shaken vigorously, tissue allowed to settle then supernatant collected. These steps were repeated to generate 4 fractions. Fractions were centrifuged at 100 *g* for 4 min and tissue integrity assessed. The crypts/villi were counted and resuspended in FOM at ~200/mL and plated out; Advanced DMEM/F12 (TFS) supplemented with 10 mM HEPES (TFS), 2 mM l-Glutamine (TFS), 50 U/mL Penicillin/Streptomycin (Merck) and 2% B27 supplement (50X; TFS). Where indicated the enteroid cultures were supplemented with 25 ng/mL EGF (Prepotech), 25 ng/mL Noggin (Enzo Life Sciences), 250 ng/mL R-spondin (R&D Systems), 100 mM Y-27632 (Cambridge Bioscience), 100 mM SB202190 (Enzo Life Sciences) and 5 mM LY2157299 (Cambridge Bioscience). Differentiation of avian enteroids occurred at 37 °C, 5% CO_2._ After 24 h the floating enteroids were transferred to wells containing fresh media and half the media was replaced every 2 days by tilting the plates. For duodenal, jejunal, caecal and quail enteroids the isolation and culture protocols were kept the same.

To seed chicken intestinal crypts/villi in growth factor reduced (GFR) Matrigel (Corning), the isolated material was resuspended in equal volumes of FOM and ice-cold GFR Matrigel to allow for 50 per 50 µL and cultured as described for mouse intestinal crypts, using FOM instead of Intesticult medium (Stemcell Technologies)^1^.

### Isolation of mouse intestinal crypts

Intestinal crypts were dissociated from mouse small intestine using Gentle Cell Dissociation Reagent (Stemcell Technologies). Crypts were used to establish enteroids by cultivation in GFR Matrigel surrounded by Intesticult medium as previously described^1^. Crypts to be cultivated without GFR Matrigel were seeded floating in Intesticult medium at ~200/mL and fresh medium was replaced every 2 days.

### Transmission electron microscopy (TEM)

Enteroids were fixed in 3% glutaraldehyde in 0.1 M sodium cacodylate buffer, pH 7.3, for 2 h and processed as described previously^4^. Ultrathin sections (60 nm) were stained in uranyl acetate and lead citrate and imaged using a JEOL JEM-1400 Plus TEM and analysed using ImageJ v1.52e (Fiji).

### Whole-mount and immunohistochemical (IHC) staining

Details of the sources, clone numbers and concentrations of the primary and secondary antibodies used for IHC are provided in Supplementary Table 1. Enteroids were fixed with 4% paraformaldehyde then blocked with 5% v/v goat serum in permeabilisation solution (0.5% v/v bovine serum albumin and 0.1% v/v Saponin in PBS) and stained with primary and secondary antibodies (Supplementary Table 1) at 4 °C. DNA was stained with 4’, 6-diamidino-2-phenylindole (DAPI; TFS) or Hoechst (TFS) and, where indicated, F-actin was visualised with Alexa Fluor conjugated Phalloidin (TFS). Enteroids were then mounted in ProLong® Diamond Antifade Mountant (TFS). Isotype and negative controls were prepared for each staining. Samples were visualised using a LSM710 or LSM880 Confocal Microscope (Zeiss) and processed using ImageJ v1.52e.

Proliferation was assessed in a 5-ethynyl-2′-deoxyuridine (EdU) incorporation assay, by using a Click-iT EdU Alexa Fluor 488 cell proliferation assay kit (Invitrogen). Enteroids were incubated with 10 μM EdU for 1 h and nuclei stained (Hoechst; TFS).

Intestinal tissue was snap frozen in liquid nitrogen, 10 µm sections were prepared on a Leica Cryostat CM1900 and mounted on Superfrost Plus slides (TFS). Tissues were fixed in 50% methanol then blocked and stained as described for staining of whole mount enteroids.

### Phagocytosis assay

Enteroids were grown to 3 and 7 days of culture and then 100 enteroids/well were seeded into 48 well plates (Corning) containing 125 μg/ml pHrodo red zymosan bioparticles (Molecular Probes) in FOM. Controls had 20 μM Cytochalasin D (Cayman Chemical) added to the media to inhibit zymosan uptake. Enteroids were incubated at 37 °C 5% CO_2_ and samples were taken at 24 and 48 h post-incubation with zymosan. Samples were fixed, stained and imaged as previously described.

### Enteroid passaging

Enteroids were mechanically disrupted by vigorous pipetting using a 5 mL syringe and 25 g × 1” needle. The dissociated enteroid fragments were centrifuged at 100 *g* for 1 min, supernatant removed and fragments re-suspended and cultured as described for chicken enteroids above, with and without listed growth factors.

### Villus and enteroid cryopreservation

Villi, immediately post-isolation, and enteroids, after 2 days of culture, were pelleted at 290 *g* for 4 min at room temperature, then resuspended in Cryostor CS10 cryopreservation medium (Stemcell Technologies) at ~1000 villi/mL. Cryovials were stored overnight at −80 °C, and then transferred to −155 °C.

To resuscitate the villi and enteroids, cryovials were thawed and mixed with 10 mL prewarmed DMEM then centrifuged at 290 *g* for 5 min at room temperature. The villi or enteroids were re-suspended and cultured as described for avian enteroids above.

### Epithelial barrier integrity

Enteroids were washed and resuspended in fluorescein isothiocyanate (FITC)-Dextran (4 kDa, 2 mg/mL in PBS) (Sigma Biosciences). Controls were treated with 2 mM EDTA in HBSS for 15 min, then washed and resuspended in FITC-Dextran solution. Enteroids were immediately imaged using a LSM880 Confocal Microscope and processed using ImageJ.

### RNA sequencing

Enteroids from three cultures were collected at 0, 3 and 7 days of culture, lysed in RLT buffer (Qiagen) containing 10 µg/mL 2-mercaptoethanol (Sigma-Aldrich) and homogenised using Qiashredder columns (Qiagen). Each culture (biological replicate) arose from 3 pooled embryos. Two duplicate plates were cultured for each biological replicate, and samples were taken from each plate as technical replicates. RNA was extracted using the RNeasy mini kit (Qiagen) according to the manufacturer’s protocol including DNase I treatment. The RNA quality and concentration was assessed using D1000 Screentape Agilent System (Agilent Technologies) then stored at −80 °C. Libraries were prepared and sequenced on Illumina Novaseq 6000 by using 150-bp paired-end sequencing. Obtained reads were trimmed for quality and to remove adaptor sequences using Cutadapt^62^. Reads after trimming were required to have a minimum length of 50 bases. Paired-end reads from Illumina sequencing were aligned to the *Gallus gallus* genome (Gallus_gallus-5.0) using STAR^63^. The annotation used for counting was the standard GTF-format annotation for that reference (annotation v91). Raw counts for each annotated gene were obtained using the featureCounts software (v1.5.2^64^). Differential gene expression analysis was performed within the Bioconductor edgeR package (v3.16.5^65^). Comparison of the embryonic enteroid transcriptome at 0, 3 and 7 days post cultivation revealed that there were no differentially expressed genes between the technical replicates (FDR < 0.05), demonstrating the consistency and reproducibility of the enteroid system. The sumTechReps function in EdgeR was used to merge technical replicates. All subsequent steps were performed on the merged samples. The raw counts table was filtered to remove genes consisting predominantly of near-zero counts, filtering on counts per million (CPM) to avoid artefacts due to library depth. Statistical assessment of differential expression was carried out with the likelihood-ratio test. Differentially expressed genes were defined as those with FDR < 0.05 and logFC > 2. Heatmaps were constructed in R using the pheatmap package (v1.0.10^66^).

### Infection of enteroids

*Salmonella enterica* subspecies enterica serovar Typhimurium strain 4/74 carrying a chromosomal pFVP25.1::gfp fusion linked to the naladixic acid resistance gene was utilised for infections of the chicken enteroids and compared to a defined mutant, ST4/74 *nal*^*R*^ *ΔprgH::kan* (kindly provided by Dr P. Vohra). This *prgH* mutant is confirmed to have reduced Type 3 secretion by analysing secretion of SipC, a *Salmonella* type III secretion system (T3SS) effector protein, and was also transformed with the plasmid pFVP25.1 which constitutively expresses GFP^67,68^. Strains were cultured overnight in Luria-Bertani (LB) broth with 50 µg/mL kanamycin (not used for wild-type ST4/74 *nal*^*R*^), 50 µg/mL ampicillin and 20 µg/mL naladixic acid at 37 °C. Wells containing 50 enteroids were inoculated with 5 × 10^4^ bacteria in antibiotic-free FOM and incubated statically at 37 °C, 5% CO_2_. Samples were collected after 30 min and 4 h of infection for analysis. Bacterial replication was measured by incubating enteroids with the *Salmonella* strains at 37 °C, 5% CO_2_ for 1 h, then high-dose gentamicin (50 µg/mL) was added to the wells for 30 min. Enteroids were washed and incubated with low-dose gentamicin (10 µg/mL) added to FOM (without Penicillin/Streptomycin). Enteroids were collected at 0, 3 and 8 h post high-dose gentamicin treatment and disrupted using steel beads in a Tissue-Lyser. Serial dilutions were plated on naladixic acid containing LB agar in duplicate and incubated at 37 °C overnight. The compared results were expressed as mean ± standard deviations (SD).

Fifty enteroids were incubated with 2 × 10^7^ PFU H1N1 virus (A/Puerto Rica/8/34 (PR8), kindly provided by Prof P. Digard) in DMEM supplemented with 50 U/mL Penicillin/Streptomycin and 50X v/v B27 at 37 °C, 5% CO_2_ for 1 h. Control cultures either had PBS or allantoic fluid from uninfected chicken eggs added to the media. The enteroids were then washed and reseeded in DMEM media supplemented with 2 μg/mL TPCK-trypsin and collected at 48 h post-wash for analysis. Supernatants were harvested at 0 and 48 h post-wash and titrated by plaque assay on MDCK cells as previously described^69^. The compared results were expressed as mean ± SD.

Frozen purified *Eimeria tenella* sporozoites (kindly provided by Dr E. Wattrang) were washed in warm DMEM and labelled with PKH67 Green Fluorescent Cell Linker kit (Sigma-Aldrich) according to manufacturer’s protocol. ~5 × 10^4^ sporozoites were added to each well containing fifty 2 day old chicken enteroids. These were incubated at 37 °C, 5% CO_2_. Fresh caecal enteroids, cultured for 2 days, were added to the cultures at 4 and 6 dpi to provide fresh epithelial cells for the merozoite stages. Enteroids were collected for analysis at 1, 2, 4, 7 and 9 dpi. Total RNA was isolated from the enteroid cultures as described above. PCR was performed using the Taq DNA Polymerase kit (Invitrogen) as described by the manufacturer and previously described *EtGAM56* PCR primers^70^.

### Statistics and reproducibility

The legends of each figure provide most details of sample sizes, numbers of replicates, number of repeats and statistics used. Data presented are mean ± SD and significant differences between samples at different time points were determined by Student’s *t*-test. Where variance was not equal between two groups, a Mann–Whitney *U*-test was used. Where results were not normally distributed and there were more than 2 groups, a Kruskal-Wallis test was used. A linear regression test was employed to model the relationship between time (h) and *Salmonella* replication (CFU). A linear mixed effects model with post hoc pairwise *t*-tests were used to model the relationship between time (day) and enteroid morphology. All measurements were recorded from distinct samples except for Fig. 1j where repeated measurements were made against the same enteroids over time i.e. dependent. Anderson-Darling normality tests were used to assess the normal distribution. Values of *T* > 2 or < −2 and *p* < 0.05 were accepted as significant (*), *p* < 0.001 as very significant (**), and *p* < 0.0001 as extremely significant (***). Unless otherwise stated, images are representative of data from at least 3 independent cultures each containing 2–3 embryos. In each avian culture of 48 wells or murine culture of 12 wells, 4 pooled wells with ~100 enteroids/well were used for each histochemistry sample. RNA sequencing data is representative of 3 independent experiments, each comprising of 2 technical replicates, each containing 3 embryos. Each RNA sample was comprised of 8 pooled wells containing ~100 enteroids/well.

### Reporting summary

Further information on research design is available in the Nature Research Reporting Summary linked to this article.

## Supplementary information

Supplementary Information

Description of Additional Supplementary Files

Supplementary Video 1

Supplementary Data 1

Reporting Summary

## Data Availability

The authors declare that all data supporting the findings in this study are available within the article, within Supplementary Data 1 file or from the corresponding author on reasonable request. The mRNA expression datasets for this study have been deposited in the European Nucleotide Archive (ENA) at EMBL-EBI under accession number PRJEB37491.
