# Supplementary material for: Inside-out chicken enteroids with leukocyte component as a model to study host–pathogen interactions
Source: Commun Biol. 2021 Mar 19;4:377. doi: 10.1038/s42003-021-01901-z (PMC7979936; doi:10.1038/s42003-021-01901-z)
Supplement: Supplementary file 1 — Supplementary Information [file 42003_2021_1901_MOESM1_ESM.pdf]

Supplementary Information

Inside-Out Chicken Enteroids with Leukocyte Component as a Model to Study Host-Pathogen Interactions

Tessa J. Nash, Katrina M. Morris, Neil A. Mabbott and Lonneke Vervelde

This file includes:

Supplementary Table 1

Supplementary Figures 1 - 4

Supplementary Table 1 Primary antibodies used for immunohistochemistry.

Target	Antibody details	Clone	Catalog number	Dilution
Mucin 5AC	Mouse anti-mucin 5AC [a]	45M1	Ab212636	20 µg/ml
Lysozyme C	Rabbit anti-lysozyme [a]	polyclonal	Ab391	20 µg/ml
Chromogranin A [SP1]	Rabbit anti-chromogranin A [b]	polyclonal	20085	1.3 µg/ml
Sox9 [phospho S181]	Rabbit anti-SOX9 [a]	polyclonal	Ab59252	2 µg/ml
Villin	Mouse anti-villin [c]	1D2C3	Sc-58897	4 µg/ml
E-Cadherin	Mouse anti-E-Cadherin [d]	36/E-Cadherin	610181	5 µg/ml
ZO-1 tight junction protein	Rabbit anti-ZO1 [a]	polyclonal	Ab216880	10 µg/ml
Virus nucleoprotein	Rabbit anti-NP [e]	polyclonal	n/a	1:1000
CD45	Mouse anti-CD45[f]	AV53	n/a	1:100
ChB6	Mouse anti-Bu-1[g]	AV20	839502	2 µg/ml
CD3	Mouse anti-CD3[h]	CT-3	820009	2 µg/ml

[a] Abcam
[b] Immunostar
[c] Santa Cruz Biotechnology
[d] BD Biosciences
[e] provided by Prof P. Digard; Noton *et al.* 2007 http://doi.org/10.1099/vir.0.82809-0
[f] Institute for Animal Health; Garceau *et al.* 2015 http://doi.org/10.1186/s12915-015-0121-9
[g] Southern Biotech; Rothwell *et al.* 1996 http://doi.org/10.1016/S0165-2427(96)05635-8
[h] Southern Biotech; Göbel *et al.* 1994 http://doi.org/10.1002/eji.1830240734

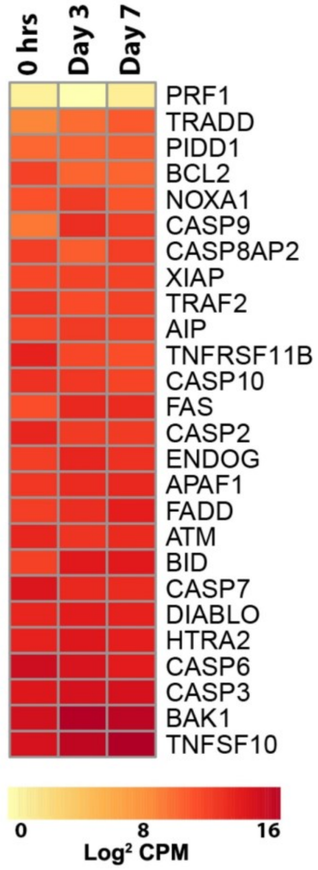

Supplementary Figure 1 | Expression of apoptosis-related genes. Gene expression in freshly isolated villi (0 h), 3 day and 7 day chicken enteroids was compared by RNA sequencing analysis. Heat maps show the expression levels (log2 counts per million reads) of a range of apoptosis-related genes. RNA sequencing data is representative of 3 independent experiments each comprising of 2 technical replicates each containing 3 embryos.

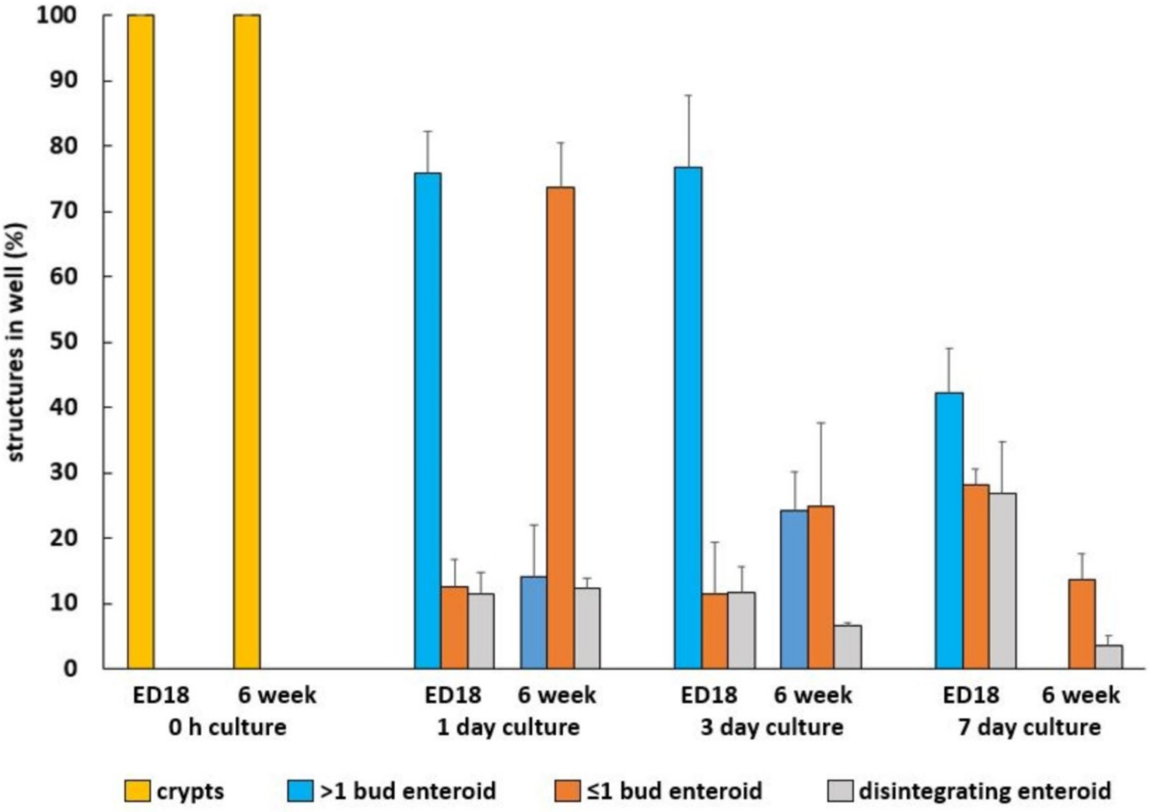

Supplementary Figure 2 | Budding and viability of enteroids derived from 6 week old chicken gut compared to enteroids derived from embryonic tissue. Floating chicken enteroids derived from 6 week chicken jejunum display substantial lower number of buds per enteroid at 1 day of culture. The viability in general in comparison to enteroids derived from ED18 small intestine is lower. Bars represent mean ± SD derived from 3 independent experiments each containing 2-3 embryos or one 6 week old chicken, 3 wells/culture.

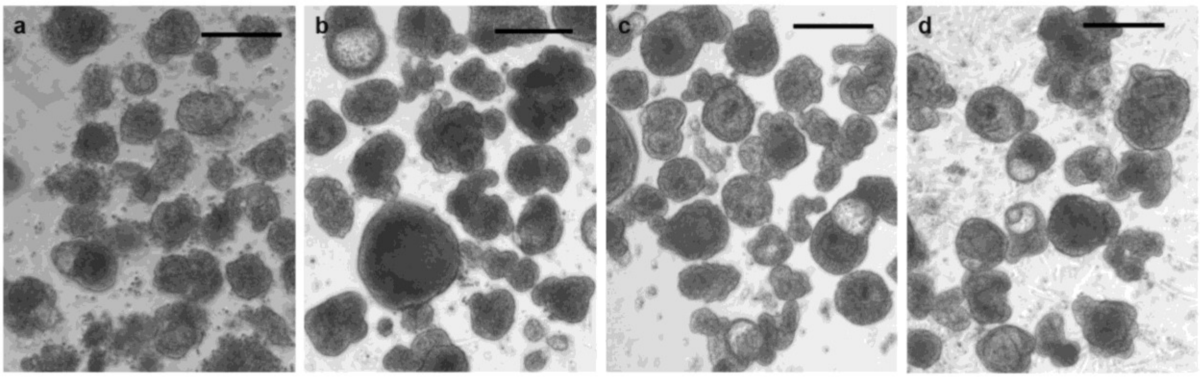

Supplementary Figure 3 | Chicken enteroid recovery from cryopreservation. **a** Cryopreserved 2 day enteroids at point of thaw and **b** after 3 days of culture post-thaw, compared to **c** freshly cultured 2 day enteroids and **d** 5 day enteroids. Images **a – d** are representative of at least 3 independent cultures each containing 2-3 embryos. Scale bar: 200 µm.

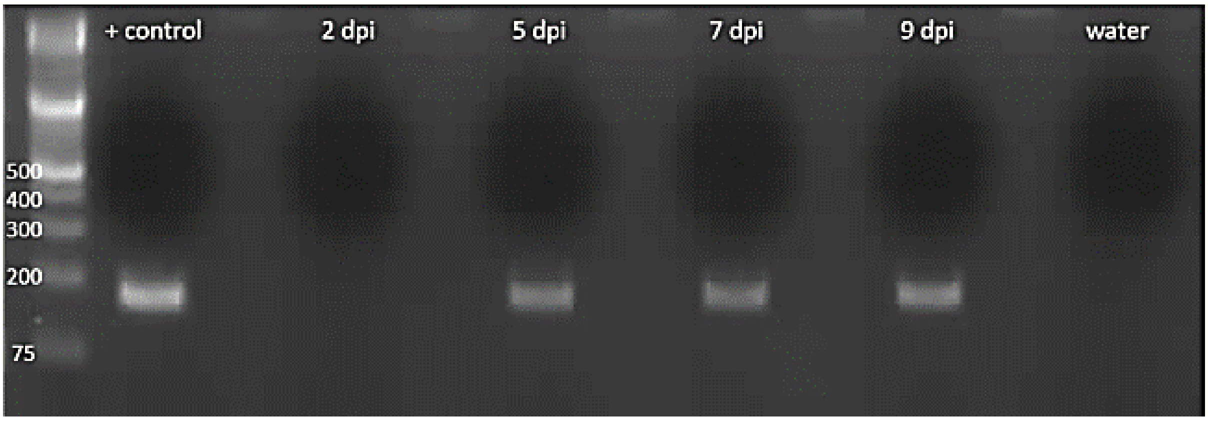

Supplementary Figure 4 | PCR of *Eimeria tenella* gamete marker *EtGAM56* in infected caecal enteroid. Positive (+) control is chicken caecal tissue from 6 dpi and 13 dpi after *in vivo* infection with *Eimeria tenella.* Caecal enteroids were infected with *Eimeria tenella* for 2 days, 5 days, 7 days and 9 days. Band at 178bp where *EtGAM56* expected in positive control as well as 5, 7 and 9 dpi. No band evident at 2 dpi or in water control.
