# Supplementary material for: Inside-out chicken enteroids with leukocyte component as a model to study host–pathogen interactions
Source: Commun Biol. 2021 Mar 19;4:377. doi: 10.1038/s42003-021-01901-z (PMC7979936; doi:10.1038/s42003-021-01901-z)
Supplement: Supplementary file 2 — Description of Additional Supplementary Files [file 42003_2021_1901_MOESM2_ESM.pdf]

Description of Additional Supplementary Files

File name: Supplementary Video 1 **- Budding of floating enteroids.**

Description: Brightfield images of floating chicken embryonic crypts were captured by time-lapse imaging using a Zeiss Live cell observer with a 10x objective lens. The enteroid culture was imaged every 5 min from 1 to 25 h of culture. The image was cropped, using Image J, to a region of interest demonstrating efficient budding of enteroids. Scale bar: 100 µm.

File name: Supplementary Data 1

Description:

Fig 1j **Raw counts of enteroid numbers and morphology over 9 days of culture.** 3 independent cultures quantified, 3 wells per culture averaged, each culture containing 2 - 3 embryos.

Fig 4m **Raw counts of bud number in enteroids derived from chicken embryonic duodenum, jejunum and caecum**. Number of buds per ED18 enteroid after 2 days of culture were manually counted. 3 independent experiments each containing 2-3 embryos, 3 - 4 wells/culture quantified.

Fig 4n **Raw counts of bud length in enteroids derived from chicken embryonic duodenum, jejunum and caecum**. Length of buds on ED18 enteroids after 2 days of culture were manually measured using Image J. 3 independent experiments each containing 2-3 embryos, 3 wells/culture were quantified.

Fig 8g **Raw CFU counts for *Salmonella* net replication assay.** Bacterial colony count from 5 independent experiments with 2-3 embryos per culture, ~800 infected enteroids post high-dose gentamicin treatment.

Fig 9d **Raw viral titers for influenza A virus strain PR8 (H1N1) plaque assay.** Plaque assay performed on 4 independent experiments, each containing 2-3 embryos and ~800 seeded enteroids/well, using supernatant at 0 and 48 hpi.
