# Supplementary material for: Inside-out chicken enteroids with leukocyte component as a model to study host–pathogen interactions
Source: Commun Biol. 2021 Mar 19;4:377. doi: 10.1038/s42003-021-01901-z (PMC7979936; doi:10.1038/s42003-021-01901-z)
Supplement: Supplementary file 5 — Reporting Summary [file 42003_2021_1901_MOESM5_ESM.pdf]

nature research

Corresponding author(s): *Double-blind peer review submissions: write DBPR and your manuscript number here instead of author names.*

Last updated by author(s): Dec 16, 2020

Reporting Summary

Nature Research wishes to improve the reproducibility of the work that we publish. This form provides structure for consistency and transparency in reporting. For further information on Nature Research policies, see our Editorial Policies and the Editorial Policy Checklist.

Statistics

For all statistical analyses, confirm that the following items are present in the figure legend, table legend, main text, or Methods section.

n/a	Confirmed	
☐	☒	The exact sample size (*n*) for each experimental group/condition, given as a discrete number and unit of measurement
☐	☒	A statement on whether measurements were taken from distinct samples or whether the same sample was measured repeatedly
☐	☒	The statistical test(s) used AND whether they are one- or two-sided<br>*Only common tests should be described solely by name; describe more complex techniques in the Methods section.*
☐	☒	A description of all covariates tested
☐	☒	A description of any assumptions or corrections, such as tests of normality and adjustment for multiple comparisons
☐	☒	A full description of the statistical parameters including central tendency (e.g. means) or other basic estimates (e.g. regression coefficient) AND variation (e.g. standard deviation) or associated estimates of uncertainty (e.g. confidence intervals)
☐	☒	For null hypothesis testing, the test statistic (e.g. *F*, *t*, *r*) with confidence intervals, effect sizes, degrees of freedom and *P* value noted<br>*Give P values as exact values whenever suitable.*
☒	☐	For Bayesian analysis, information on the choice of priors and Markov chain Monte Carlo settings
☒	☐	For hierarchical and complex designs, identification of the appropriate level for tests and full reporting of outcomes
☒	☐	Estimates of effect sizes (e.g. Cohen's *d*, Pearson's *r*), indicating how they were calculated

Our web collection on statistics for biologists contains articles on many of the points above.

Software and code

Policy information about availability of computer code

Data collection	Reads were trimmed using Cutadapt (version cutadapt-1.9.dev2). The reference used for mapping was the Gallus gallus genome (version 5) from Ensembl. The annotation used for counting was the standard GTF-format annotation for that reference (annotation version 91). Reads were aligned to the reference genome using STAR (version 2.5.2b) specifying paired-end reads and the option --outSAMtype BAM Unsorted. Reads were assigned to features of type 'exon' in the input annotation grouped by gene_id in the reference genome usingbfeatureCounts (version 1.5.1). Reads were normalised using the weighted trimmed mean of M-values method, passing 'TMM' as the method to the calcNormFactors method of Bioconductor edgeR package (version 3.16.5). Images were collected using Zen 2012 Blue Edition (version 10.0.18362) and Zen Black (version 8.0.0.273)
Data analysis	Differential analysis was carried out with Bioconductor edgeR package (version 3.16.5). The sumTechReps function in EdgeR (version 3.16.5) was used to merge technical replicates. Heatmaps were constructed in R using the pheatmap package (v. 1.0.10). Image J 1.52e (version Java 1.8.0_172)was used to measure enteroid bud length. Microsoft Excel was used to create bar graphs (version 16.0.5005.1000). Statistics were performed using Minitab (version 17.1.0)

For manuscripts utilizing custom algorithms or software that are central to the research but not yet described in published literature, software must be made available to editors and reviewers. We strongly encourage code deposition in a community repository (e.g. GitHub). See the Nature Research guidelines for submitting code & software for further information.

Data

Policy information about availability of data

All manuscripts must include a data availability statement. This statement should provide the following information, where applicable:

- Accession codes, unique identifiers, or web links for publicly available datasets
- A list of figures that have associated raw data
- A description of any restrictions on data availability

The authors declare that all data supporting the findings in this study are available within the article and its Supplementary Information files or from the corresponding author on reasonable request. The mRNA expression datasets for this study have been deposited in the European Nucleotide Archive (ENA) at EMBLEBI under accession number PRJEB37491 (https://www.ebi.ac.uk/ena/data/view/PRJEB37491).

Field-specific reporting

Please select the one below that is the best fit for your research. If you are not sure, read the appropriate sections before making your selection.

- [x] Life sciences
- [ ] Behavioural & social sciences
- [ ] Ecological, evolutionary & environmental sciences

For a reference copy of the document with all sections, see nature.com/documents/nr-reporting-summary-flat.pdf

Life sciences study design

All studies must disclose on these points even when the disclosure is negative.

Sample size	Sample sizes varied from 3 repeated organoid cultures to counting 800 individual organoids, sample sizes depended on the type of measurement to be conducted. The lowest sample size of 3 biological replicates was used for the RNA sequencing analysis and was limited by the cost of the sequencing however of each biological replicate a technical replicate was included to demonstrate reproducibility.
Data exclusions	No data were excluded from the analyses
Replication	To verify replication of the organoid cultures, three biological replicates (each replicate is a pool of 3 embryos) were plated in duplicate resulting in 3 biological replicates and 2 technical replicates per culture with sampling occurring at 3 different time points. All samples were submitted for RNAsequencing. The low number of DEG between technical replicates confirmed the excellent level of replication and the highly significant clustering of the biological replicates on principal component analysis further confirmed the validation of the newly developed organoid cultures.
Randomization	Randomization was not relevant for our study. We mostly used 18 day old chicken embryos, and the eggs were randomly collected and set by the university facility supplying the eggs. The intestinal tracts of the other animals (mice quail and 9 week old birds) were also supplied by the university animal facilities and not selected for this experiment but shared between researchers to limit the number of animals (3Rs). Not a single animal was exposed to experimental procedures (solely tissue donors).
Blinding	Blinding was not relevant for our studies.

Reporting for specific materials, systems and methods

We require information from authors about some types of materials, experimental systems and methods used in many studies. Here, indicate whether each material, system or method listed is relevant to your study. If you are not sure if a list item applies to your research, read the appropriate section before selecting a response.

Materials & experimental systems

n/a	Involved in the study	
[ ]	[x]	Antibodies
[x]	[ ]	Eukaryotic cell lines
[x]	[ ]	Palaeontology and archaeology
[ ]	[x]	Animals and other organisms
[x]	[ ]	Human research participants
[x]	[ ]	Clinical data
[x]	[ ]	Dual use research of concern

Methods

n/a	Involved in the study	
[x]	[ ]	ChIP-seq
[x]	[ ]	Flow cytometry
[x]	[ ]	MRI-based neuroimaging

Antibodies

Antibodies used	Details of the sources, clone numbers and concentrations of the primary and secondary antibodies used for IHC are provided in Supplementary Table 1. This table contains the target antigen detected, species in which antibody is generated, clone name, catalog

	number, dilution used and literature reference describing the validation.
Validation	Literature references to describe the validation of the antibodies are given in supplementary table 1 or can be found on the suppliers website linked to the catalogue number.

Animals and other organisms

Policy information about studies involving animals; ARRIVE guidelines recommended for reporting animal research

Laboratory animals	Species and age ranges and provider (university animal facilities) are given in the M&M. In each figure legend the specific age of the animal is clearly defined. Sex has not been determined of the chicken embryos and the quail, the mice were all female.<br>Hy-Line Brown chickens, CSF1R-eGFP transgenic chickens, quail (Coturnix coturnix), C57BL/6 mice
Wild animals	n/a
Field-collected samples	n/a
Ethics oversight	All animals were housed in premises licensed under a UK Home Office Establishment License in full compliance with the requirements of the Animals (Scientific Procedures) Act 1986 and with approval from The Roslin Institute Animal Welfare Ethics Review Board. The review board approved the breeding use of transgenic chickens but the animals were not exposed to experimental procedures and therefore they did not provide further advice on the protocols. The animals were culled according to UK Home Office guidance specific for the age of the animal.

Note that full information on the approval of the study protocol must also be provided in the manuscript.
